# Prostatic duct adenocarcinoma—A challenging variant of prostate cancer in a low‐resource setting

**DOI:** 10.1002/ccr3.9557

**Published:** 2024-11-04

**Authors:** Ronald Okidi, Nakazibwe Hajarah, Derrick Mukurasi, Emmanuel Obonyo, Emmanuel Makai, Daniel Lajul, Moses Odongkara, Tracy Bamutorana, BridgetRose Aguti Omagino, Isaac Okello

**Affiliations:** ^1^ Department of Surgery Lacor Hospital Gulu Uganda; ^2^ Faculty of Medicine, Department of Surgery Gulu University Gulu Uganda; ^3^ Central and Southern Africa College of Surgeons of East Arusha Tanzania; ^4^ Kalongo, Department of Surgery Dr Ambrosoli Hospital Kalongo Uganda; ^5^ Medical Teams International (MTI) Lacor Hospital Gulu Uganda

**Keywords:** androgen deprivation therapy, chemotherapy, metastases, prostate cancer, prostatic ductal adenocarcinoma, radiotherapy

## Abstract

This case emphasizes the challenges in diagnosing and treating Prostatic Duct Adenocarcinoma, especially in resource‐limited settings. Early and accurate diagnosis is crucial for better patient outcomes, but limited access to advanced diagnostics and specialized treatments significantly hinders the effective management of this aggressive form of prostate cancer.

## 1 INTRODUCTION

Prostate cancer is becoming an increasingly serious health issue in Africa, with rising rates of illness and death. This trend is especially evident in Uganda.^1^ Prostatic ductal adenocarcinoma (PDA), a rare and aggressive form of prostate cancer, originates in the large primary periurethral prostatic ducts, and poses a higher risk of progression and spreading to other organs.^2^, ^3^ Although PDA is the second most common subtype of prostatic carcinoma, it makes up only a small percentage of cases, with acinar adenocarcinoma accounting for over 90% of all primary prostatic carcinomas.^4^ PDA is frequently found alongside the more common acinar carcinoma, indicating that it may sometimes develop in conjunction with the prevalent acinar adenocarcinoma subtype.^2^

## 2 CLINICAL PRESENTATION

PDA often displays distinct clinicopathological and radiological characteristics that can be difficult to detect before surgery.^5^ Patients usually exhibit urinary obstructive symptoms, hematuria, and are frequently diagnosed during transurethral resection.^6^ These tumors typically grow as outward lesions into the urethra, particularly around the verumontanum.^6^, ^7^, ^8^ PDA is marked by gross hematuria or urinary obstructive symptoms such as delayed urination, nocturia, and dribbling.^8^, ^9^ Ductal adenocarcinoma cases often present with advanced disease and are poorly differentiated exhibiting unique metastatic patterns, commonly spreading to both bone and visceral organs. Ranasinghe et al. noted that while bone metastases in PDA are similar to acinar adenocarcinoma, lung metastases occur more frequently, rising from 23.2% in de novo cases to 44.2% post‐treatment.^10^, ^11^ Vinjamoori et al. further identified atypical metastases in PDA, with the lungs and pleura affected in 40%, the liver in 37%, and supradiaphragmatic lymph nodes in 34% of cases.^12^ Bergamin et al. also observed that PDA often metastasizes to unusual sites, such as the lungs, even at low prostate‐specific antigen (PSA) levels.^13^ These findings emphasize PDA's aggressive and unpredictable metastatic behavior.

## 3 INVESTIGATIONS

PDA exhibits variable serum PSA levels and is more aggressive than acinar prostate cancer.^14^, ^15^ Patients often present with normal digital rectal examination (DRE) and PSA levels, delaying diagnosis, and PDA primarily affect older men, usually appearing in the periurethral or peripheral zones of the prostate.^2^

PDA shows distinct characteristics, such as lower PSA secretion, less ETS‐related gene (ERG) and phosphatase and tension homolog expression, higher P16 and P53 expression, and reduced steroid‐related markers, indicating different tumorigenesis mechanisms compared to conventional prostate cancer.^16^ Despite normal DRE results, multiparametric magnetic resonance imaging (MRI) is the preferred diagnostic method, though PDA can be hard to detect on some MRI sequences.^17^, ^18^, ^19^

Biopsy and cytohistologic studies are essential for accurate diagnosis, as PDA can mimic other prostatic conditions like High‐grade prostatic neoplasia (HGPIN), intraductal prostatic carcinoma, hyperplastic variants of prostate cancer, cribriform Gleason Pattern 4 acinar adenocarcinoma, and metastases.^17^ PDA's rarity and frequent coexistence with acinar adenocarcinoma complicate histological identification. Lower serum PSA levels in PDA compared to acinar cancer can hinder its detection.^10^

## 4 MANAGEMENT

Early treatment improves the prognosis for patients with PDA.^20^ However, the best management approach for PDA is unclear due to its rarity and limited data.^21^, ^22^ Typically, a multimodal approach is used, including chemotherapy, radiotherapy, and radical prostatectomy (RP), tailored to the individual patient.^23^ PDA often exhibits extracapsular extension, positive margins, and seminal vesicle involvement. Pure PDA has a higher risk of local recurrence after prostatectomy compared to mixed PDA. Conventional therapies like hormonal and radiotherapy are less effective, making local control crucial, and serum PSA levels are unreliable for staging or predicting recurrence.^2^ RP and radiotherapy are generally preferred for localized disease, while androgen deprivation therapy (ADT) and chemotherapy are used for metastatic disease. There is no consensus on the optimal treatment for PDA.^4^

## 5 CLINICAL CASE SUMMARY

### 5.1 History

43‐year‐old male who presented with a one‐year history of straining associated with hesitancy, dribbling, weak stream feeling of incomplete bladder emptying, and then eventually developed gross hematuria throughout micturition and urine retention. He also reported general body weakness, progressive weight loss, and low‐grade fevers.

On examination, he was mildly pale and had a blood pressure of 118/65 mmHg and a pulse rate of 59 beats per minute. The abdominal was soft nontender, with no masses or organs palpable, with a suprapubic catheter in situ and a digital rectal exam, and had a grossly enlarged, firm prostate gland with no surface nodularity, and no area of tenderness.

### 5.2 Investigations

Transrectal ultrasonography (TRUS) revealed a significantly enlarged prostate with a volume of 530 cc, displaying an uneven and irregular texture. The patient had a PSA level of 0.26 ng/mL, a granulocyte counts of 4500 cells/mm^3^, a hemoglobin level of 12.5 g/dL, and a platelet count of 167,000 cells/ mm^3^. Additionally, the serum creatinine level was 0.8 mg/dL, and the urea level was 25 mg/dL.

### 5.3 Management

**FIGURE 1:**
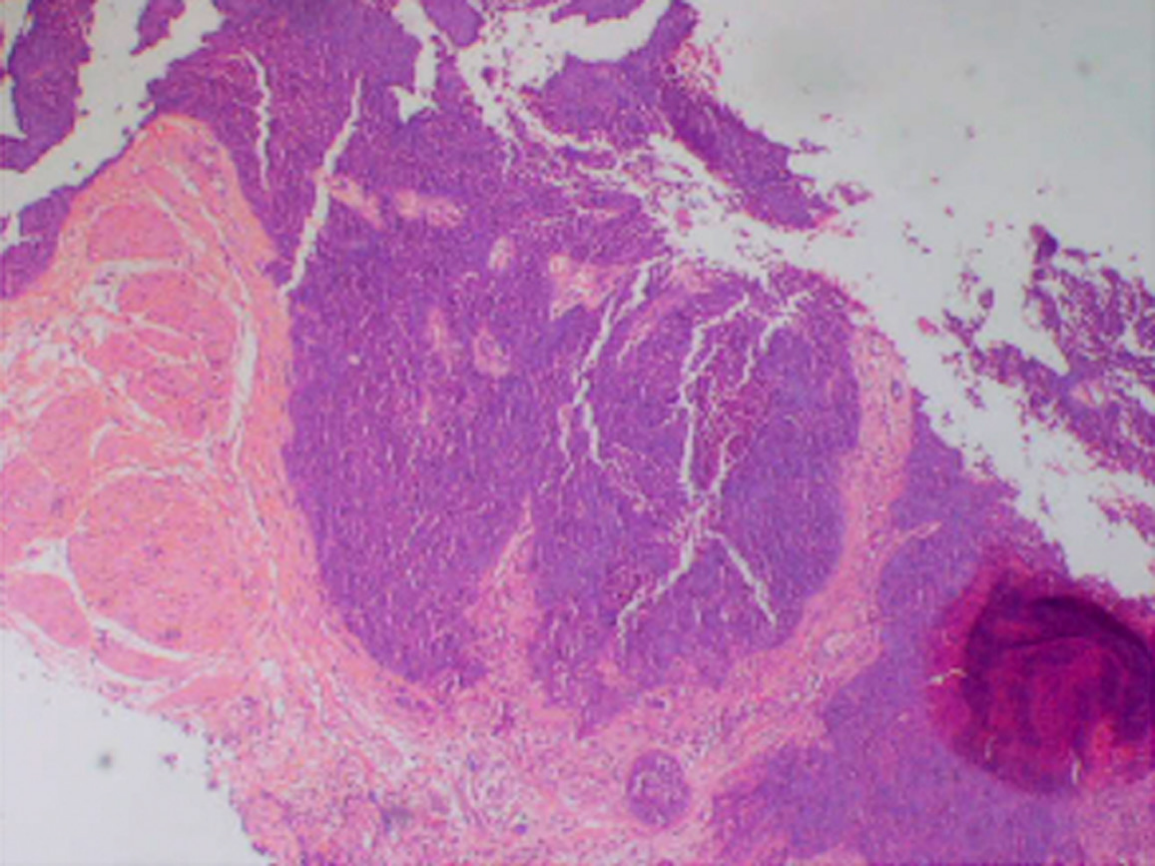
The biopsies show infiltrating neoplastic pleomorphic cells growing in sheets consistent with a Gleason 10 ductal adenocarcinoma of the prostate. There is perineural invasion. There is no evidence of extraprostatic extension in the biopsies. The tumor involves approximately 100% of the total tissue sample.

A transvesical open simple prostatectomy was attempted, and the prostate was found adherent to the pelvic side walls, a biopsy was done and it confirmed prostatic duct adenocarcinoma with a Gleason score of 10 (5 + 5) (Figure 1). An abdominal ultrasonogram revealed liver metastases, a chest X‐ray was normal.

Surgical ADT (orchiectomy), Abiraterone and Prednisolone were then initiated, and a channel transurethral resection of prostate was performed that temporarily relieved his obstructive symptoms. However, he continued to deteriorate despite initial cancer treatment and was referred to Uganda Cancer Institute—Mulago for further cancer care where he died within the month of referral from metastatic visceral disease.

## 6 CASE DISCUSSION

This case highlights the significant hurdles in diagnosing and treating PDA, particularly in settings with limited resources. The patient presented with classic symptoms of urinary obstruction, which are also common in benign prostate enlargement, making early diagnosis difficult.^2^, ^3^, ^5^, ^6^, ^24^ Additionally, he experienced episodes of blood in the urine (hematuria), a well‐documented symptom of PDA.^8^, ^9^ Unfortunately, this patient's case also exemplified the aggressive nature of PDA. Many PDA cases, like this one, present with advanced disease at diagnosis, with this patient having metastasis to the liver.^10^, ^12^, ^13^, ^19^ The tumor's infiltrative growth pattern, which we couldn't confirm by endoscopic visualization, further complicated the situation. His enlarged prostate volume of 530 mL disqualified him from a standard endoscopic procedure due to the limitations of available equipment.^6^, ^7^, ^8^ The lack of access to advanced imaging like multiparametric MRI added another layer of difficulty in definitively diagnosing the disease.^18^, ^19^

Initially, the patient's normal PSA level and PSA density led to a misdiagnosis of benign prostatic hyperplasia (BPH). This resulted in an attempt at a standard surgical procedure “open transvesical simple prostatectomy” to remove the prostate, but the procedure proved a futile choice and impossible due to the cancerous nature of the enlarged gland. This not only failed to treat the cancer but also caused unnecessary complications for the patient.^10^, ^24^

Unfortunately, the patient couldn't receive the most effective treatment “RP” due to the advanced stage of the cancer and the limited availability of chemo‐radiation therapy in his region. These treatments are only offered at a single specialized cancer center in the entire country. Given the concerning progression of the disease despite starting ADT, the patient was eventually placed on a combined therapy of abiraterone and prednisolone.^21^, ^22^ Sadly, even this treatment regimen proved ineffective.^2^

## 7 CONCLUSION

This case emphasizes the challenges in diagnosing and treating PDA, especially in resource‐limited settings. Early and accurate diagnosis is crucial for better patient outcomes, but limited access to advanced diagnostics and specialized treatments significantly hinders effective management of this aggressive form of prostate cancer.

## AUTHOR CONTRIBUTIONS

**Ronald Okidi:** Conceptualization; supervision; writing – original draft; writing – review and editing. **Nakazibwe Hajarah:** Writing – original draft; writing – review and editing. **Derrick Mukurasi:** Writing – original draft. **Emmanuel Obonyo:** Resources; writing – original draft. **Emmanuel Makai:** Resources; writing – original draft. **Daniel Lajul:** Resources; writing – original draft. **Moses Odongkara:** Writing – original draft. **Tracy Bamutorana:** Writing – original draft. **BridgetRose Aguti Omagino:** Resources; writing – original draft. **Isaac Okello:** Conceptualization; writing – original draft; writing – review and editing.

## FUNDING INFORMATION

None.

## CONFLICT OF INTEREST STATEMENT

The authors declare no conflicts of interest.

## ETHICS STATEMENT

Ethical approval was obtained from Lacor Hospital Institutional Research and Ethics Committee (LHIREC) and the hospital administration issued clearance to report this case.

## CONSENT

The patient provided written informed consent for the publication of his case.

## Data Availability

Data will be provided upon reasonable written request to the corresponding author.
